# Rxivist.org: Sorting biology preprints using social media and readership metrics

**DOI:** 10.1371/journal.pbio.3000269

**Published:** 2019-05-21

**Authors:** Richard J. Abdill, Ran Blekhman

**Affiliations:** 1 Department of Genetics, Cell Biology, and Development, University of Minnesota, Minneapolis, Minnesota, United States of America; 2 Department of Ecology, Evolution, and Behavior, University of Minnesota, St. Paul, Minnesota, United States of America

## Abstract

Preprints have arrived. In increasing numbers, researchers across the life sciences are embracing the once-niche practice, shaking off decades of reluctance and posting hundreds of papers per week to preprint servers, sharing their findings with the community before embarking on the weary march through peer review. However, there are limited methods for individuals sifting through this avalanche of research to identify the preprints that are most relevant to their interests. Here, we describe Rxivist.org, a website that indexes all preprints posted to bioRxiv.org, the largest preprint server in the life sciences, and allows users to filter and sort papers based on download metrics and Twitter activity over a variety of categories and time periods. In this work, we hope to make it easier for readers to find relevant research on bioRxiv and to improve the visibility of preprints currently being read and discussed online.

## Abstract 2

This Community Page article describes Rxivist.org, a new website that indexes all preprints posted to bioRxiv.org and allows users to filter and sort papers based on download metrics and Twitter activity over a variety of categories and time periods.

## Introduction

A preprint is a publicly available academic paper that has not yet been published in a peer-reviewed journal. Though the acceptance and popularity of preprints took longer to take root in the life sciences than in fields such as physics and mathematics [1], more than 215,000 authors have posted preprints to bioRxiv.org [2], the website that now houses more biology preprints than all other major preprint servers combined [3]. The exponential growth of biology preprints has far outstripped even the largest pre-internet attempts [4] at circulating unrefereed publications: In the 1960s, the National Institutes of Health operated one such program, which mailed 2,561 different “memos” over the course of 6 years [5]. BioRxiv publishes that number of preprints every 5 weeks and now houses more than 47,000 papers across 27 disciplines [2], available not just to a rarefied cohort of academic subscribers but everyone with access to the web. Although less than 200 preprints were posted per month in late 2015, a quick glance at a dozen titles per day is no longer sufficient to keep up with all of the new research appearing online. BioRxiv (pronounced "Bio Archive") offers a conservative set of options for viewing these submissions: A standard text search includes the option to view the latest papers matching a search term, and preprints are broken down into 27 "subject areas" (i.e., "cancer biology," "bioinformatics," "immunology," and so on) that can be listed in reverse-chronological order. Email alerts also offer the option to receive notifications about new preprints matching search criteria. Despite these conveniences, the rapid (and expanding) rate of submissions is making the task of parsing these papers an increasingly impractical proposition—in the neuroscience category—bioRxiv's largest—447 preprints were posted just in March 2019 [2]. Although bioRxiv provides download data about each preprint, there is no way to use that information when searching.

The evaluation of these download counts and other “altmetrics” [6] is difficult to contextualize across and within fields (see Discussion). New metrics can also reinforce new incarnations of the “Matthew Effect,” a “rich get richer” dynamic in which famous scientists receive more attention (and citations) for their work [7]. Still, download metrics and Twitter activity present an interesting opportunity to organize preprints using metrics less arbitrary than chronology, as bioRxiv does. Using metadata continually collected about the full corpus of bioRxiv preprints, we built Rxivist.org (pronounced "Archivist"), a website enabling users to search, filter, and sort preprints based on download metrics and the number of Twitter messages linking to it. We hope this tool will be useful for researchers throughout the life sciences who either have too many preprints to read or are unfamiliar with the medium and are looking for somewhere to start.

## Results

The Rxivist application is made of 2 pieces: an application programming interface (API) that provides preprint data in JavaScript Object Notation (JSON) format, and a Python-based website that uses this data to build lists of preprints that conform to a user's search parameters. These services provide human- or machine-readable access to browsable data on preprint altmetrics and a list of the preprints currently being discussed on Twitter.

### Preprint listings

Users visiting the homepage will find the default search parameters already filled in, displaying the 20 most discussed preprints on Twitter.com since the beginning of the previous day. New preprints are pulled from bioRxiv 6 times per day, along with updated Twitter activity reflecting which ones are currently being discussed (see Methods). This means a visitor who checks Rxivist.org once per day should always find new content. In the default view, preprints with more than 110 tweets in the current day are marked with a "fire" icon to signify a paper with an exceptional number of tweets in that day. This level was selected by sorting recent daily Twitter data going back to September 2018 and determining what value would have resulted in 35 percent of nonweekend days having a “hot” paper.

**Fig 1:**
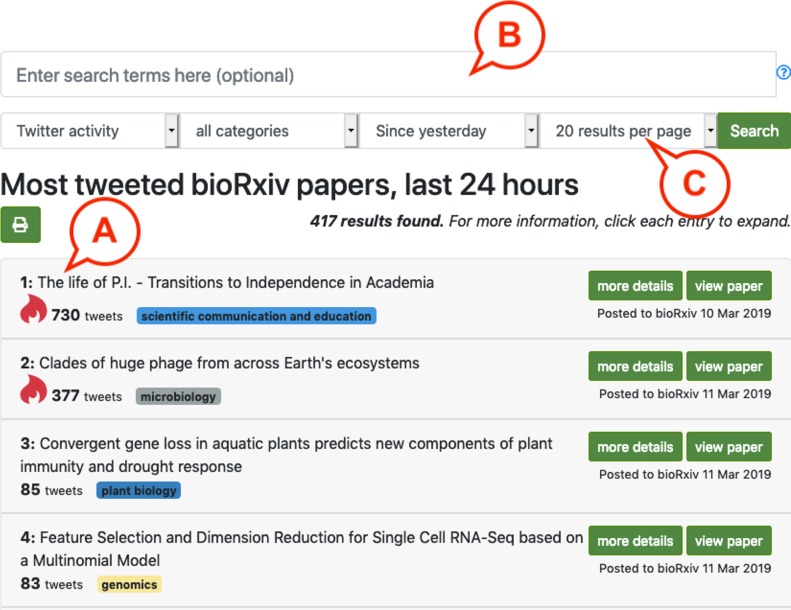
The top of the Rxivist.org homepage. The default results settings for the Rxivist.org homepage, including the search box and top results based on Twitter metrics (A) on 1 March 2019. Below the text search field (B) are 4 drop-down menus (C) that provide the other available parameters—which metric to use in the ranking process, whether to limit results to a particular category, the timeframe in which the metrics should be limited, and how many results to return at one time.

The search box (Fig 1) provides several options for modifying this search. Results can be restricted by category, a parameter that can be combined with a modified timeframe: Twitter data can be used based on the previous 1, 7, or 30 days or viewed without any time restrictions, which incorporates tweet counts dating back to early 2017. For example, a user could request the most discussed microbiology preprints of the last week (https://rxivist.org/?category=microbiology&timeframe=week).

Twitter metrics provide a strong signal for capturing the online day-to-day discussions about preprints, and daily readers will find the Twitter search results to generate a more dynamic list of recommendations. Monthly download data sourced from the bioRxiv website provide a longer-term sorting method that has lower resolution but may have a more direct connection to the actual readership of a particular preprint. Because downloads are only specified in monthly intervals and each preprint's metrics are updated in the Rxivist index about once every 2 weeks, users can choose from a smaller set of timeframes, either starting at the beginning of the previous month, year-to-date totals, or all-time downloads. Category-level filtering is still available for these lists, so a user could ask, e.g., for the most downloaded bioinformatics preprints of the current year (https://rxivist.org/?metric=downloads&category=bioinformatics&timeframe=ytd).

**Fig 2:**
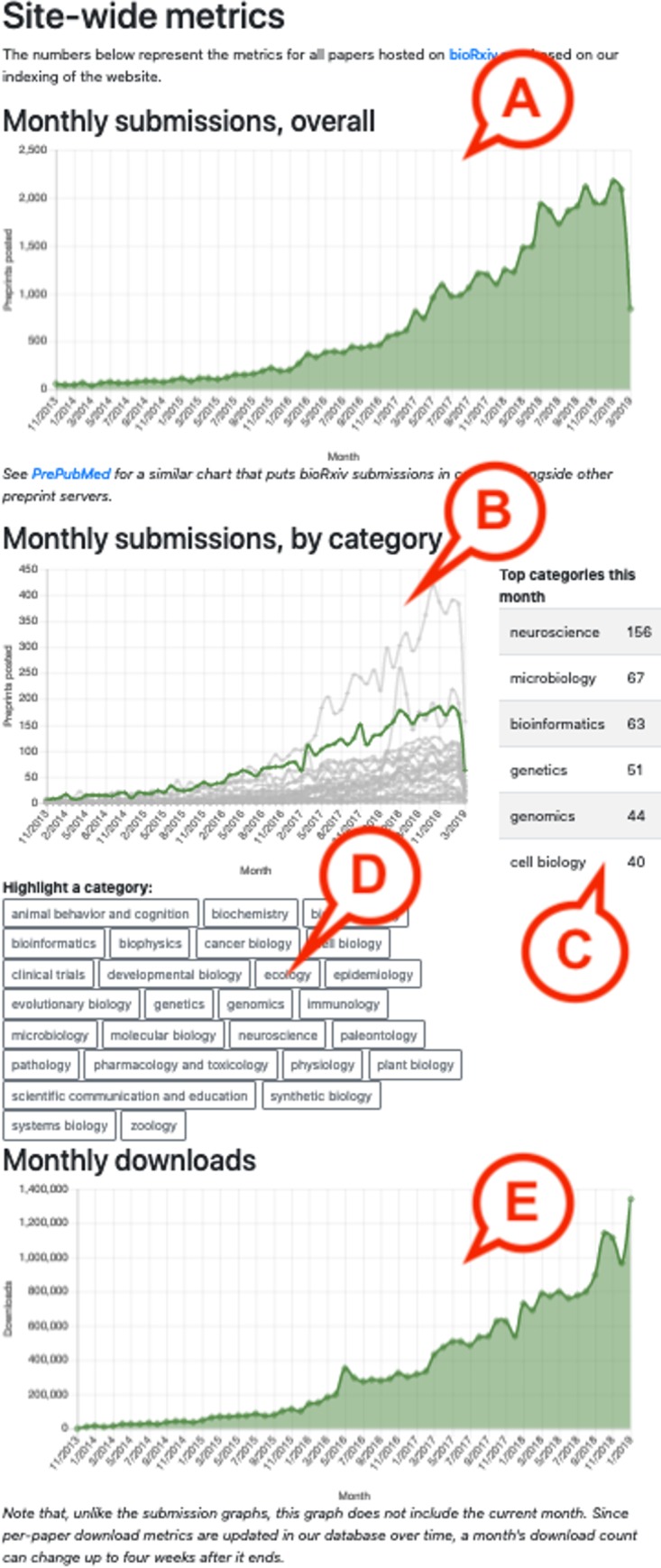
The summary metrics page. The summary metrics page (https://rxivist.org/stats) includes a chart of submissions per month (A), plus a similar chart broken down by category (B) and a table showing the categories that have received the most preprints in the current month (C). Users can highlight individual categories in the chart using buttons (D). The final graph (E) plots total monthly downloads.

Several other pages segment the data in a different way: There is a page listing the most downloaded preprints of 2018 (https://rxivist.org/top/2018), which lists 25 preprints posted in that year and orders them based on downloads through December 2018. Similar pages are available for papers dating back to 2013 (https://rxivist.org/top/2013). In addition, a summary page (https://rxivist.org/stats) visualizes overall metrics for the bioRxiv collection, including monthly totals for submissions and downloads (Fig 2).

### Detailed profiles

**Fig 3:**
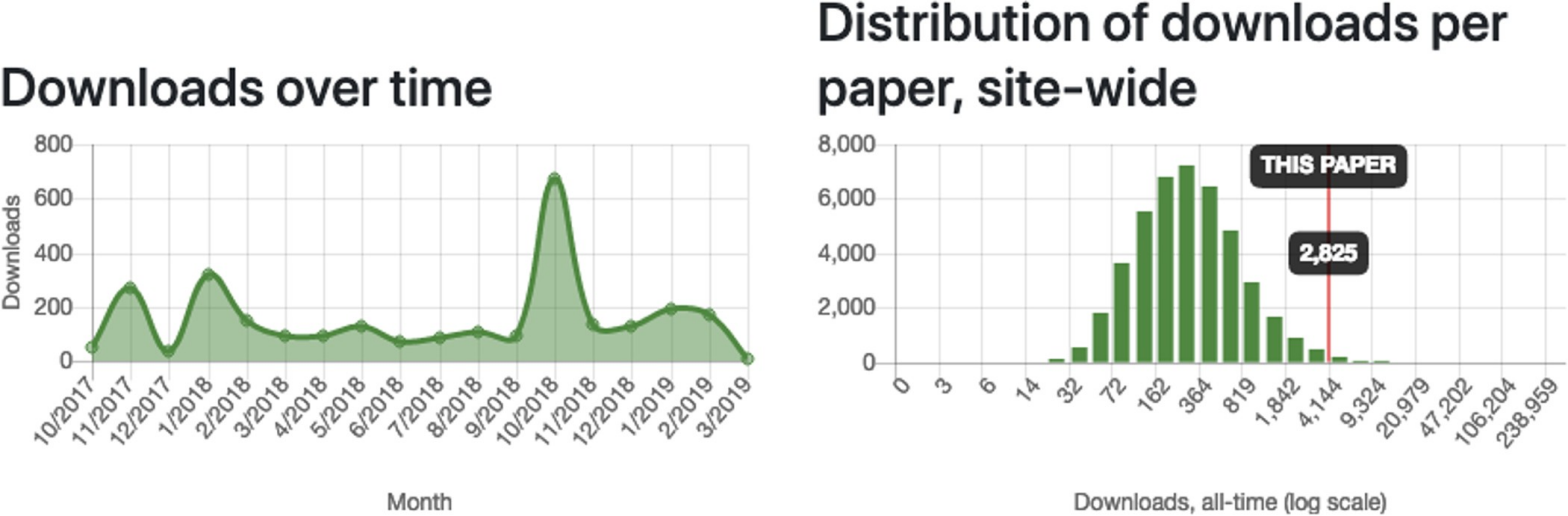
Preprint-level metrics visualization. A screenshot showing typical graphs of download metrics displayed on an Rxivist profile page for an individual preprint. The left plot shows a single paper's downloads (y-axis) per month (x-axis), and the right plot is a histogram (with a log scale on the x-axis) of total downloads per preprint of all preprints on bioRxiv, including an indication of which bin includes the preprint in question. This example is from the page at https://rxivist.org/papers/10.1101/210294.

In addition to generating sorted lists of preprints, the data scraped from bioRxiv is also used to create profile pages for each preprint and author that has been indexed. Each preprint has a profile page specifying its title, abstract, digital object identifier (DOI), and 2 plots of longitudinal download data: one visualizes downloads over time and the other shows where that preprint's total download count compares to all others (Fig 3). Whereas the histogram compares the paper to all other preprints on bioRxiv, each profile also includes download rankings in multiple timeframes, including all-time rankings both site-wide and within the category to which it was first posted. In-category rankings are probably the most informative of these comparisons, because download counts vary widely between categories [8].

Each paper profile page also includes an embedded visualization of data from Altmetric.com, a commercial service that indexes mentions of academic works (including preprints) within an expansive collection of social media platforms. Altmetric, like Crossref, does not offer a publicly available method of browsing these results.

Each preprint's profile also includes a full author list that links to individual profile pages for each author. Author profiles are more complex and combine data from all preprints attributed to that author based on name or open researcher and contributor identifier (ORCID ID; see Methods). Each author profile includes basic information, such as their name and the institutional affiliation specified on their most recent preprint, plus download rankings that compare each author based on the cumulative downloads of all their preprints, which is also displayed. Each author is given a site-wide rank for all-time downloads and also receives a ranking in each category to which they've posted a preprint. There is also a histogram (similar to the one in Fig 3) that shows the distribution of total downloads per author and indicates where the author in question falls. Beneath the download information is a list of all preprints for which the individual is listed as an author, plus the individual paper rankings for each.

### API

The Rxivist platform also includes an API for programmatic interaction with the data—rather than responding with a hypertext markup language (HTML) document, the server responds to API requests by sending back JSON-formatted data that is more easily parsed by applications.

The "search" endpoint (https://api.rxivist.org/v1/papers) accepts many of the same options presented on the website—e.g., one could request the most downloaded preprints in the "biophysics" category that include the word "NMR" (https://api.rxivist.org/v1/papers?q=NMR&metric=downloads&category=biophysics); the response includes basic information about the results (total results, how many are displayed in the current response, and so on), plus an array of preprints. Each preprint in this array is a JSON object specifying properties including the preprint's title, uniform resource locator (URL), DOI, abstract, and an array of authors that indicates each author's full name and their unique identifier in the Rxivist database.

There is a separate endpoint to get more specific information about a particular preprint. Each paper is given a unique, stable identification (ID) number in the Rxivist database; adding that number to the end of the "search" endpoint (i.e., https://api.rxivist.org/v1/papers/25770) will return information about only that paper: the same data available in the search response, plus download rankings reflecting that preprint's standing in the list of all preprints. Rather than requesting a preprint's details using its Rxivist ID number—an arbitrary integer assigned when a preprint is first indexed—a paper can also be requested using its DOI: for the example above, using the URL https://api.rxivist.org/v1/papers/10.1101/096727 would return the same data.

The "paper details" endpoint also includes the ORCID ID and institutional affiliation of each author, in the order they are listed on the preprint. The preprint's ID can also be passed to the "downloads" endpoint (i.e., https://api.rxivist.org/v1/downloads/25770) to get monthly download metrics for a given paper. This response also includes the number of abstract views recorded by bioRxiv, which is currently not displayed on the Rxivist website.

Similarly, passing an author's ID number to the "authors" endpoint (i.e., https://api.rxivist.org/v1/authors/345042) will return a JSON object that includes the author's name, ORCID ID, email addresses (as submitted to bioRxiv), and the person's most recently observed institutional affiliation. The response also includes a list of all papers associated with the author, plus the rankings of each paper and of the author's cumulative downloads. A request to the "authors" endpoint that does not specify an author ID (https://api.rxivist.org/v1/authors) will return a list of 200 authors ordered by their total downloads and whether they are tied with 1 or more other authors with that total. Passing a category parameter to this URL (i.e., https://api.rxivist.org/v1/authors?category=bioinformatics) will return the same results but using the download totals only of papers posted to that category.

There are also several endpoints for metadata on the Rxivist index itself. They are fully documented online (https://rxivist.org/docs), but the main endpoint here is the "total entities" endpoint (https://api.rxivist.org/v1/data/stats), which returns the total number of preprints and authors in the database, plus a count of how many preprints in the index are missing important information such as an abstract, category, or date of posting. It also lists how many papers have "outdated" information, currently defined as a paper that has not received updated download metrics in the last 4 weeks.

## Discussion

### Next steps

The future feature set of Rxivist.org is currently constrained primarily by development resources: With enough time and contributions from the open-source community (see Methods), there are a number of possibilities for future improvements, and we would gladly welcome contributions from external developers. Although the website is currently rendered mostly on the server using Python 3, it would likely benefit from being refactored into a client-side JavaScript application. The most frequently requested enhancement is a more diverse selection of email newsletters: Currently, a weekly email lists the 20 most tweeted stories of the week, but readers have expressed interest in more specifically tailored information—say, the most tweeted preprints in genetics, genomics, and microbiology, or the most discussed preprints that include a keyword of interest. A system for allowing users to log into Rxivist accounts could enable the registration of relevant categories or terms, and improved email automation could tailor updates to individuals. User logins could also allow the customization of the Rxivist homepage and sorting algorithms that account for user preferences, similar to the approach taken by papr (https://jhubiostatistics.shinyapps.io/papr/), which recommends bioRxiv preprints based on user input. This approach is taken by some of the many websites [9] that act as an overlay for arxiv.org, a large scientific preprint server founded in 1991 that specializes in fields such as physics, mathematics, and computer science: Arxivsorter (https://www.arxivsorter.org) and arxivist (http://arxivist.com, of which we were unaware when we named our website) make recommendations based on user endorsements; Arxiv Sanity Preserver (http://arxiv-sanity.com) does the same and also provides a “top hype” list ranked by Twitter activity.

We also hope to develop a more nuanced selection option for “front-page” preprints: a section highlighting preprints by first-time bioRxiv authors, possibly, or a system that accounts for varying levels of interest across different fields. There are also interesting possibilities related to addressing skewed dynamics within the collection that could result in unnecessarily homogenous results—e.g., the huge (and accelerating) increase in neuroscience preprints [8] could crowd out the fields that are either smaller or less enthusiastic about preprints. Differences in the social media influence of famous researchers could also be accounted for: Imagine a lab (Lab A) that has posted 12 preprints, all of which received 100 tweets on the day they were posted. In contrast, Lab B has posted 12 preprints, which received an average of 3 tweets on the day they were posted. If labs A and B each post a new preprint, is it more notable that Lab A got 100 tweets (again) or that Lab B got 70 tweets (for the first time)?

Until recently, the contents of bioRxiv preprints was available only in PDF files, which present unique challenges to parsing and processing. However, bioRxiv has recently started incorporating the full text and figures of all preprints available in multiple machine-readable formats [10]. Although the process is still ongoing, this presents many opportunities for better characterizing preprints using natural language processing techniques, particularly regarding future recommendation engines that could have functionality similar to that of services such as Google Scholar (https://scholar.google.com), Scholarfy (https://www.scholarfy.net), and Meta (https://meta.org).

### Using altmetrics

Rxivist uses tweet counts and download numbers to surface preprints that are being discussed and downloaded. There are downsides to using these numbers but none that are not also present in traditional publishing: bioRxiv download counts have been shown to be vulnerable to manipulation [11] but so have citation counts [12]. Discussions of online platforms are frequently fraught with unexamined assumptions about factors such as race and gender [13–15], but the entrenched hierarchies of traditional publishing have well-studied inequalities of their own (e.g., [16]), and traditional measurements of impact have been blown by the shifting winds of bias since they were developed [17,18]. Every new metric also risks a reincarnation of the “Matthew Effect” [7]—authors with lots of downloads and existing "microcelebrity" in their field [19] may just end up getting more downloads. However, there is no evidence that the risk in this case is any greater than the effect's current form relating to notoriety and citations, as observed more than a half-century ago.

We are optimistic about the utility of exploring nontraditional metrics. Our current metrics for the “success” of a paper relies mostly on citation count: Journals decide which papers are published, and those papers use their References sections to award each other points. In contrast, a single tweet linking to a single preprint represents the short-circuiting of a century of checks put in place to control how—and by whom—research is shared.

A 2012 report by Wouters and Costas [20] on tracking scholarly impact drew a distinction between “technologies of narcissism”—to wit, tools “that allow the researcher to make some sort of limited self-assessment with respect to the response to his/her work”—and “technologies of control,” which are held to higher standards of data quality and “indicator reliability.” Although Wouters and Costas are optimistic about the potential for altmetrics, the value of download rankings falls short of a “technology of control,” which could be used for professional assessment and performance evaluation. What we are left with is a “technology of narcissism”: Although it may be helpful to enable authors to determine how their preprint compares to others in their field, practical context is still missing from this summary metric—as they state in the report, “To which dimension of science does the number of tweets relate?”

Certainly, the broader implications of altmetrics on scholarly evaluation remain unsettled. But the design of the Rxivist homepage is predicated on the notion that “number of tweets” is not meant as a “technology of control,” that download count and Twitter activity are themselves interesting metrics, even if we cannot directly link them to broader conclusions. bioRxiv users now have a way to view which research is being discussed in their fields from a perspective other than their own. Even the savviest Twitter users in science aren’t connected to every community and subfield—now, readers can click through to see who’s discussing those preprints and what they’re saying. Rxivist.org is intended as a tool to explore the altmetrics of preprints, which are at least partially driven by forces independent of traditional publishing [21]. While readers are narrowing down their reading lists, we hope it will help elevate work that they may not otherwise have seen.

## Methods

We provide a detailed explanation of the data collection and processing systems behind the Rxivist website in a paper analyzing long-term trends in bioRxiv preprints [8]. Because programmatic access to bioRxiv content (via routes such as data dumps or an API) is currently unavailable, we built a Python-based web crawler that parses the bioRxiv website, detects newly posted preprints, and stores metadata about each one in a PostgreSQL database: title, authors, submission date, category, DOI, and abstract, plus the email address and institutional affiliation of each author, and, if the preprint has been published, its new DOI and the journal in which it appeared. The bioRxiv page for each preprint is then revisited about once every 2 weeks to retrieve updated download metrics and less frequently to check on its publication status. Although the web crawler is sensitive to formatting changes on the bioRxiv website, it was designed to be flexible in this regard and can be quickly modified to accommodate new markup; we intend to maintain this system for the foreseeable future, provided bioRxiv continues to allow web scraping and their site design remains practical to interpret.

The web crawler also calls the Crossref Event Data API [22] 6 times per day to retrieve any tweets referencing entities with a DOI that has a prefix matching the one used for all bioRxiv preprints (10.1101). This data is stored as a daily tweet count for each unique DOI in the database, used for the Twitter-based sorting. There are occasionally temporal gaps in the Twitter coverage, and researchers have found hints of missing data in the Crossref database [23,24]. However, so far we have not encountered any serious problems with the service, which is free to use and supported by responsive developers. Crossref also uses a sophisticated collection system that finds “events” (in our case, Twitter mentions) of entities with a registered DOI by searching not just for that DOI but any related links that they are able to trace back to that entity—so tweeting a link directly to the bioRxiv PDF file for a preprint, e.g., has the same effect on observed Twitter activity as tweeting a link to that preprint’s bioRxiv webpage, or a link to another tweet that links to the preprint [22].

The website itself is built using Python 3 and the Bottle web framework [25], which builds the web pages based on data retrieved from a JSON-based Rxivist API, also built using Bottle, that is also documented both on the website (https://rxivist.org/docs) and in our previous paper [8]. The website and API are both deployed using Docker containers, which simplifies dependency management and enables the applications to be quickly launched in other environments such as local workstations.

The code for all components of this system is stored on GitHub (see Data Availability), which we also use for bug-tracking and project management. We would encourage anyone interested in contributing to look in those repositories for outstanding issues that are accessible to developers of many skill levels.

### Data availability

There are multiple web links to resources related to this project:

- The Rxivist application is available on the web at https://rxivist.org and via Gopher at gopher://origin.rxivist.org.
- The source for the web crawler and API is available at https://github.com/blekhmanlab/rxivist.
- The source for the Rxivist website is available at https://github.com/blekhmanlab/rxivist_web.
- Snapshots of the Rxivist database are generated about once per month and are available in a versioned archive hosted by Zenodo, available at https://doi.org/10.5281/zenodo.2529922.

**Box. Interacting with Rxivist**

There are multiple ways to access the preprint rankings from Rxivist.org:

- On the web: https://rxivist.org
- Weekly newsletter of highly discussed preprints: https://rxivist.org/newsletter
- API: https://rxivist.org/docs
- Monthly database snapshots: https://doi.org/10.5281/zenodo.2529922

## Abbreviations

API: application programming interface
DOI: digital object identifier
HTML: hypertext markup language
ID: identification
JSON: JavaScript Object Notation
ORCiD: open researcher and contributer identifier
URL: uniform resource locator
